# Supplementary material for: Epigenetic signatures of asthma: a comprehensive study of DNA methylation and clinical markers
Source: Clin Epigenetics. 2024 Nov 2;16:151. doi: 10.1186/s13148-024-01765-0 (PMC11531182; doi:10.1186/s13148-024-01765-0)
Supplement: Supplementary file 3 — Additional file3 (docx 5,925 KB) [file 13148_2024_1765_MOESM3_ESM.docx]

**Supplementary Tables and Figures:**

| PC | Eigen Value | % Variance | Cumulative % |
| --- | --- | --- | --- |
| PC1 | 4.4832 | 35.27% | 35.27% |
| PC2 | 2.4844 | 19.54% | 54.81% |
| PC3 | 1.5834 | 12.46% | 67.26% |
| PC4 | 1.2396 | 9.75% | 77.01% |
| PC5 | 1.0671 | 8.39% | 85.41% |
| PC6 | 0.7696 | 6.05% | 91.46% |
| PC7 | 0.5435 | 4.28% | 95.74% |
| PC8 | 0.2794 | 2.20% | 97.94% |
| PC9 | 0.1555 | 1.22% | 99.16% |
| PC10 | 0.1071 | 0.84% | 100.00% |
| Total | 12.7125 |  |  |

**Supplementary Table 1.** Table showing PC eigen values and % of total variance captured.

| PC | Inflation |
| --- | --- |
| PC1 | 0.999737038 |
| PC2 | 0.894699702 |
| PC3 | 0.861547269 |
| PC4 | 1.034053431 |
| PC5 | 1.021476296 |
| PC6 | 0.749147555 |
| PC7 | 0.805103148 |
| PC8 | 1.003658406 |
| PC9 | 0.843756081 |
| PC10 | 0.848921965 |

**Supplementary Table 2.** EWAS results for time point 1 with inflation values.

**Supplementary Table 3.** Independent attachment with a complete list of significant CpGs for all 10 timepoint 1 EWASs.

**Supplementary Table 4.** Independent attachment with a complete list of significant CpGs for each of the 10 timepoint 2 EWASs.

| snp_chr | snp_pos | ref | alt | reffrq | info | rs | snp_pval | effalt | N | cpg | cpg_chr | cpg_mapinfo | cpg_estimate | cpg_pval |
| --- | --- | --- | --- | --- | --- | --- | --- | --- | --- | --- | --- | --- | --- | --- |
| chr11 | 76270683 | A | G | 0.645075 | 1 | 11:76270683 | 1.49E-10 | 0.08471681 | 67341 | cg02046836 | 11 | 75550064 | -0.01168842 | 3.16E-11 |
| chr11 | 76299194 | G | T | 0.512605 | 1 | 11:76299194 | 2.90E-15 | 0.1050031 | 67341 | cg02046836 | 11 | 75550064 | -0.01168842 | 3.16E-11 |
| chr11 | 76301316 | C | T | 0.617355 | 1 | 11:76301316 | 3.23E-12 | 0.09592278 | 67341 | cg02046836 | 11 | 75550064 | -0.01168842 | 3.16E-11 |
| chr11 | 76301375 | C | T | 0.617005 | 1 | 11:76301375 | 8.40E-12 | 0.0961933 | 67341 | cg02046836 | 11 | 75550064 | -0.01168842 | 3.16E-11 |
| chr15 | 67441997 | T | C | 0.698821 | 1 | 15:67441997 | 3.03E-14 | 0.1094348 | 67341 | cg23515090 | 15 | 68092384 | -0.007250445 | 5.83E-09 |
| chr15 | 67442596 | C | T | 0.763772 | 1 | 15:67442596 | 8.81E-16 | 0.117968 | 67341 | cg23515090 | 15 | 68092384 | -0.007250445 | 5.83E-09 |
| chr15 | 67444747 | C | T | 0.505252 | 1 | 15:67444747 | 1.73E-12 | -0.08952885 | 67341 | cg23515090 | 15 | 68092384 | -0.007250445 | 5.83E-09 |
| chr15 | 67446785 | G | A | 0.503151 | 1 | 15:67446785 | 9.76E-13 | -0.08880695 | 67341 | cg23515090 | 15 | 68092384 | -0.007250445 | 5.83E-09 |
| chr15 | 67447452 | C | T | 0.503093 | 1 | 15:67447452 | 1.56E-12 | -0.08800783 | 67341 | cg23515090 | 15 | 68092384 | -0.007250445 | 5.83E-09 |
| chr15 | 67448899 | A | G | 0.697946 | 1 | 15:67448899 | 5.58E-14 | 0.1036299 | 67341 | cg23515090 | 15 | 68092384 | -0.007250445 | 5.83E-09 |
| chr15 | 67449660 | A | G | 0.697946 | 1 | 15:67449660 | 2.47E-14 | 0.1034242 | 67341 | cg23515090 | 15 | 68092384 | -0.007250445 | 5.83E-09 |
| chr15 | 67450305 | A | G | 0.762722 | 1 | 15:67450305 | 2.85E-15 | 0.1157078 | 67341 | cg23515090 | 15 | 68092384 | -0.007250445 | 5.83E-09 |
| chr15 | 67450893 | A | G | 0.697946 | 1 | 15:67450893 | 5.83E-14 | 0.1035278 | 67341 | cg23515090 | 15 | 68092384 | -0.007250445 | 5.83E-09 |
| chr15 | 67458152 | G | A | 0.700222 | 1 | 15:67458152 | 2.04E-13 | 0.1004536 | 67341 | cg23515090 | 15 | 68092384 | -0.007250445 | 5.83E-09 |
| chr15 | 67464013 | A | G | 0.720472 | 1 | 15:67464013 | 5.28E-12 | 0.09549112 | 67341 | cg23515090 | 15 | 68092384 | -0.007250445 | 5.83E-09 |
| chr15 | 67467541 | T | C | 0.510621 | 1 | 15:67467541 | 1.88E-11 | -0.08491053 | 67341 | cg23515090 | 15 | 68092384 | -0.007250445 | 5.83E-09 |
| chr15 | 67468285 | A | G | 0.740721 | 1 | 15:67468285 | 3.76E-14 | 0.1190431 | 67341 | cg23515090 | 15 | 68092384 | -0.007250445 | 5.83E-09 |

**Supplementary Table 5.** Summary table of the GWAS SNPs identified by Demenais et. al. to reside within 1 MB of a CpG identified at timepoint 1.

**Supplementary Table 6.** Independent attachment with a complete list of significant CpGs for all 10 timepoint 2 EWASs.

| PC | Inflation |
| --- | --- |
| PC1 | 1.027943459 |
| PC2 | 0.979550307 |
| PC3 | 0.999929682 |
| PC4 | 1.000268796 |
| PC5 | 0.775029681 |
| PC6 | 0.949333881 |
| PC7 | 0.952948417 |
| PC8 | 1.004404304 |
| PC9 | 0.999975322 |
| PC10 | 1.02109527 |

**Supplementary Table 7.** EWAS results for time point 2 with inflation values.

| Genome_Build | TP1_IlmnID | TP1_CHR | TP1_MAPINFO | TP1_estimate | TP1_pval | TP1_PC | TP2_IlmnID | TP2_CHR | TP2_MAPINFO | TP2_estimate | TP2_pval | TP2_PC |
| --- | --- | --- | --- | --- | --- | --- | --- | --- | --- | --- | --- | --- |
| 37 | cg01043901 | 13 | 43061678 | -0.0061855 | 1.23E-08 | PC7 | cg04217496 | 13 | 43119835 | 0.003371794 | 4.51E-08 | PC7 |
| 37 | cg04983687 | 16 | 88558223 | -0.0269112 | 1.64E-12 | PC7 | cg09247061 | 16 | 88571844 | 0.005722604 | 2.14E-08 | PC2 |
| 37 | cg08940169 | 16 | 88540241 | -0.0130795 | 2.54E-09 | PC7 | cg09247061 | 16 | 88571844 | 0.005722604 | 2.14E-08 | PC2 |
| 37 | cg12077754 | 2 | 75089669 | -0.0128193 | 2.36E-08 | PC7 | cg19359099 | 2 | 75060770 | -0.000276636 | 1.97E-09 | PC3 |

**Supplementary Table 8.** The CpGs from timepoint 1 that reside within 100 kb of a CpG identified at timepoint 2.

|  | Survey 1 | Survey 2 |
| --- | --- | --- |
| No | 207 (65.1%) | 219 (61.7%) |
| Yes | 111 (34.9%) | 136 (38.3%) |
| Total | 318 | 355 |
| Response Rate (%) | 74.8% | 83.5% |

**Supplementary Table 9.** Questionnaire responses for two surveys asking about asthma status prior to study enrollment for the initial 425 individuals.

| **Asthma Survey 1 and 2 Overlap** | | | | |
| --- | --- | --- | --- | --- |
|  | | Survey 2 | | Total |
|  |  | no | yes |  |
| Survey 1 | no | 151 | 21 | 172 |
|  | yes | 13 | 86 | 99 |
| Total | | 164 | 107 | 271 |

**Supplementary Table 10.** Comparison of survey responses from individuals who responded to both surveys.

| PC | Non-outliers | Outliers |
| --- | --- | --- |
| PC8 | 265 | 54 |
| PC9 | 266 | 53 |
| PC10 | 272 | 47 |

**Supplementary Table 11.** Frequencies of outliers for principal components 8, 9, and 10. Outlier status was determined using thresholds of Q1 – (3 x IQR) and Q3 + (3 x IQR).


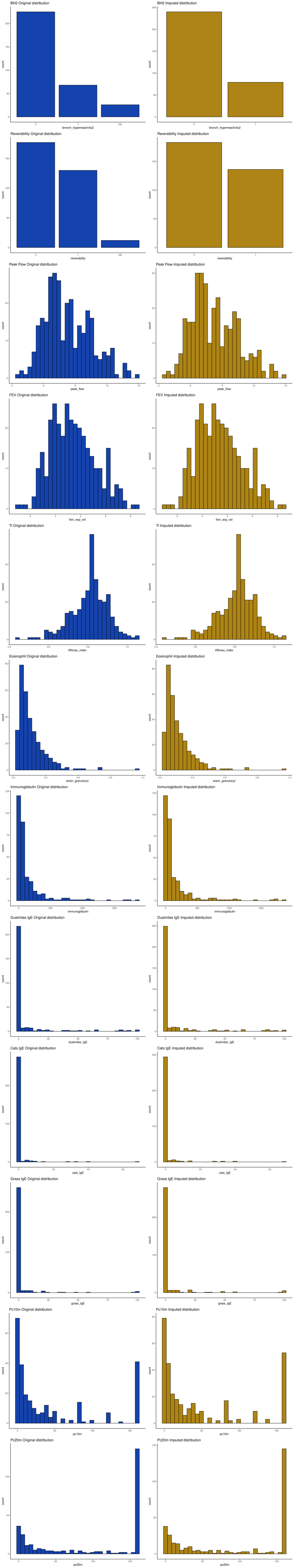

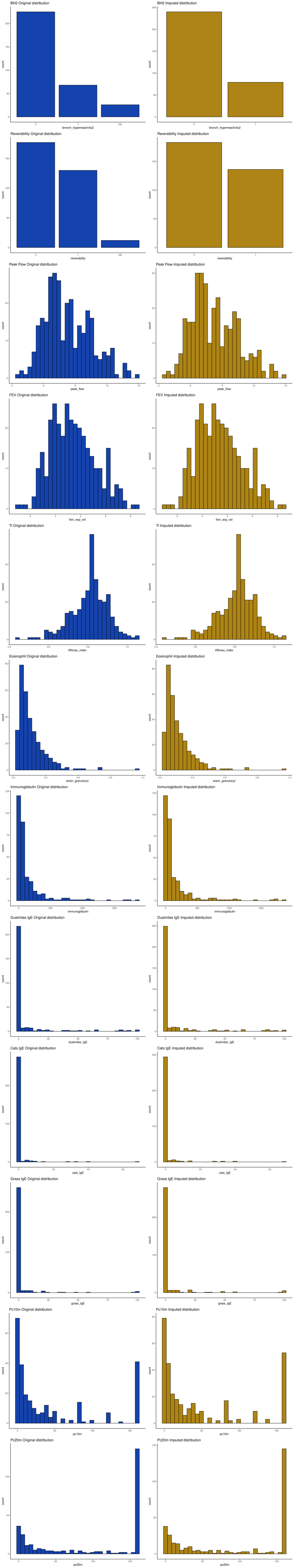


**Supplementary Figure 1.** Distribution plots of the imputed clinical markers of asthma prior to (blue) and after imputation (yellow).

**Supplementary Figure 2.** Distribution plots of the individual principal component scores for each PC.

**Supplementary Figure 3.** EWAS results calculated the 10 different sets of PC scores from the first timepoint. The red line indicates the significance threshold set via a Bonferroni correction.


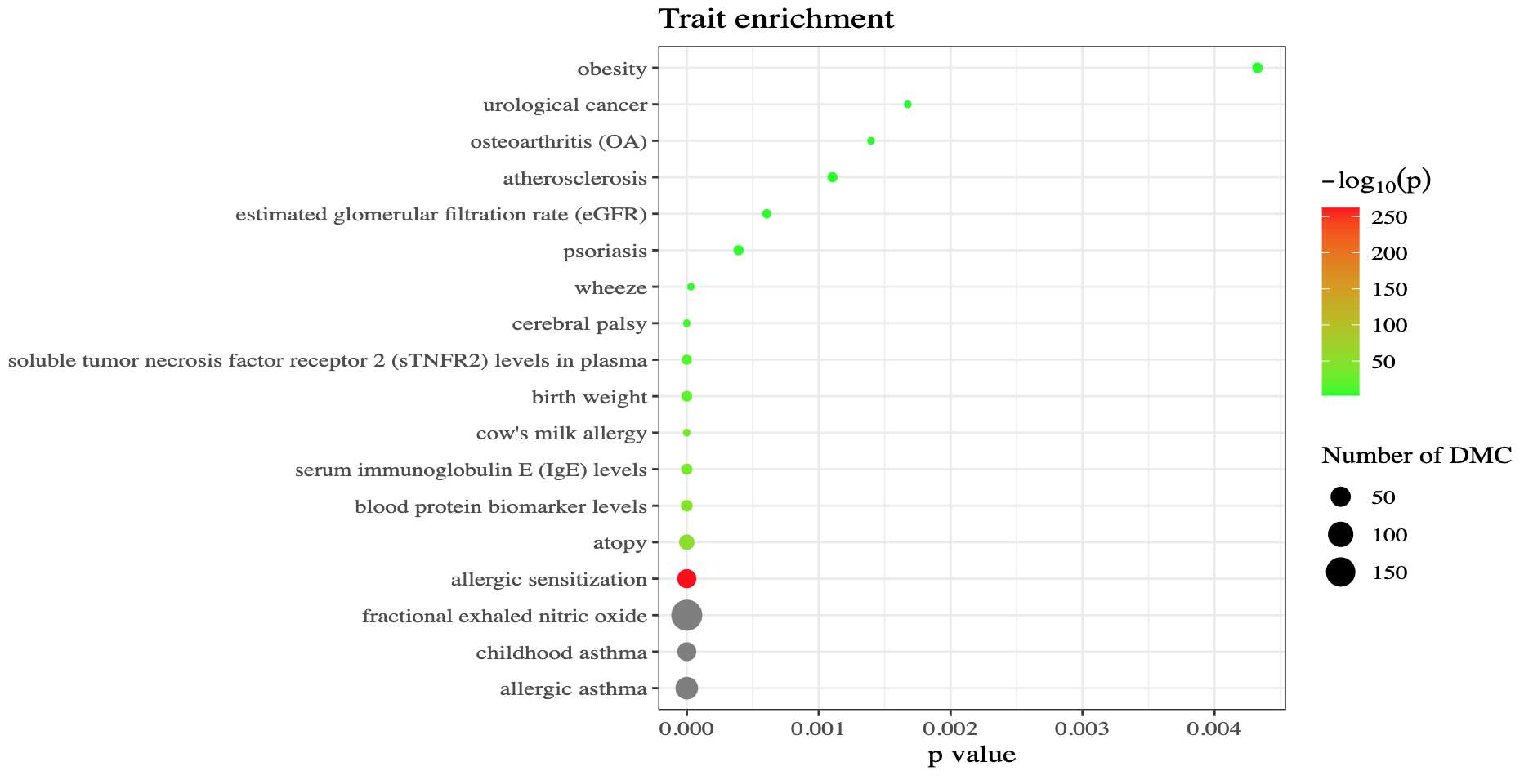


**Supplementary Figure 4.** EWAS atlas enrichment analysis of the 204 CpGs identified at TP1 from PC7. The EWAS Atlas job can be found via this job ID: 2a37ab12d2785eaa0d564e062cff5f96.

**Supplementary Figure 5.** Enrichment results from the eFORGE database (separate PDF included for better visualization).

**Supplementary Figure 6.** EWAS results calculated the 10 different sets of PC scores from the second timepoint. The red line indicates the significance threshold set via a Bonferroni correction.


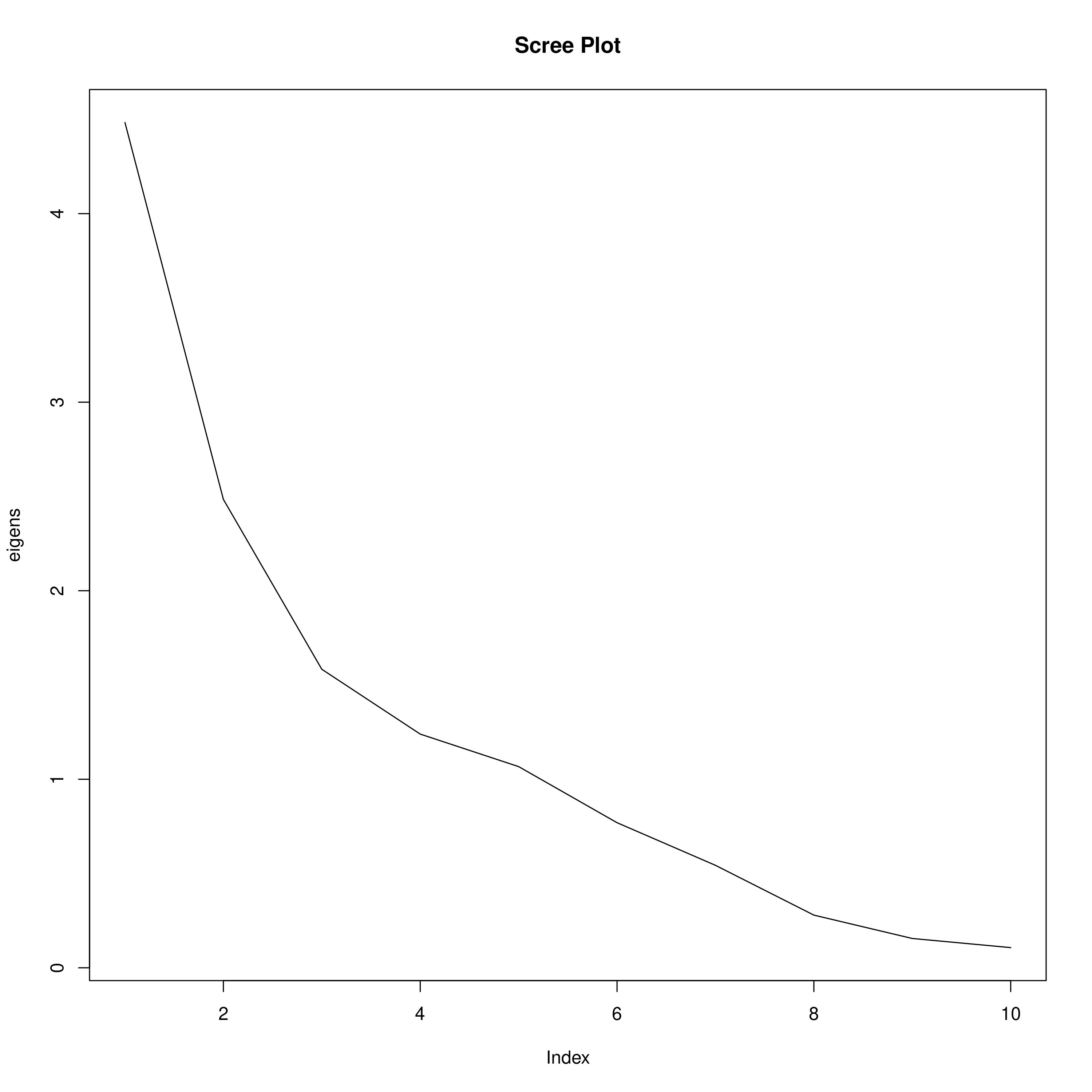


**Supplementary Figure 7.** Scree plot showing the raw eigen values for each of the 10 principal components included.
